# Is Lipid Metabolism of Value in Cancer Research and Treatment? Part II: Role of Specialized Pro-Resolving Mediators in Inflammation, Infections, and Cancer

**DOI:** 10.3390/metabo14060314

**Published:** 2024-05-29

**Authors:** Muhammad Usman Babar, Ala F. Nassar, Xinxin Nie, Tianxiang Zhang, Jianwei He, Jacky Yeung, Paul Norris, Hideki Ogura, Anne Muldoon, Lieping Chen, Stephania Libreros

**Affiliations:** 1 Department of Pathology, Yale University, New Haven, CT 06520, USA; 2 Vascular Biology and Therapeutic Program, Yale University School of Medicine, New Haven, CT 06520, USA; 3 Department of Immunobiology, Yale University, West Haven, CT 06520, USA; 4 Sciex, 500 Old Connecticut Path, Framingham, MA 01701, USA; 5 Department of Microbiology, Hyogo Medical University, Kobe 678-1297, Japan

**Keywords:** lipid mediators, resolution, docosahexaenoic acid, efferocytosis, cancer, respiratory inflammation, ischemia

## Abstract

Acute inflammation is the body’s first defense in response to pathogens or injury that is partially governed by a novel genus of endogenous lipid mediators that orchestrate the resolution of inflammation, coined specialized pro-resolving mediators (SPMs). SPMs, derived from omega-3-polyunstaturated fatty acids (PUFAs), include the eicosapentaenoic acid-derived and docosahexaenoic acid-derived Resolvins, Protectins, and Maresins. Herein, we review their biosynthesis, structural characteristics, and therapeutic effectiveness in various diseases such as ischemia, viral infections, periodontitis, neuroinflammatory diseases, cystic fibrosis, lung inflammation, herpes virus, and cancer, especially focusing on therapeutic effectiveness in respiratory inflammation and ischemia-related injuries. Resolvins are sub-nanomolar potent agonists that accelerate the resolution of inflammation by reducing excessive neutrophil infiltration, stimulating macrophage functions including phagocytosis, efferocytosis, and tissue repair. In addition to regulating neutrophils and macrophages, Resolvins control dendritic cell migration and T cell responses, and they also reduce the pro-inflammatory cytokines, proliferation, and metastasis of cancer cells. Importantly, several lines of evidence have demonstrated that Resolvins reduce tumor progression in melanoma, oral squamous cell carcinoma, lung cancer, and liver cancer. In addition, Resolvins enhance tumor cell debris clearance by macrophages in the tumor’s microenvironment. Resolvins, with their unique stereochemical structure, receptors, and biosynthetic pathways, provide a novel therapeutical approach to activating resolution mechanisms during cancer progression.

## 1. Introduction

**Figure 1:**
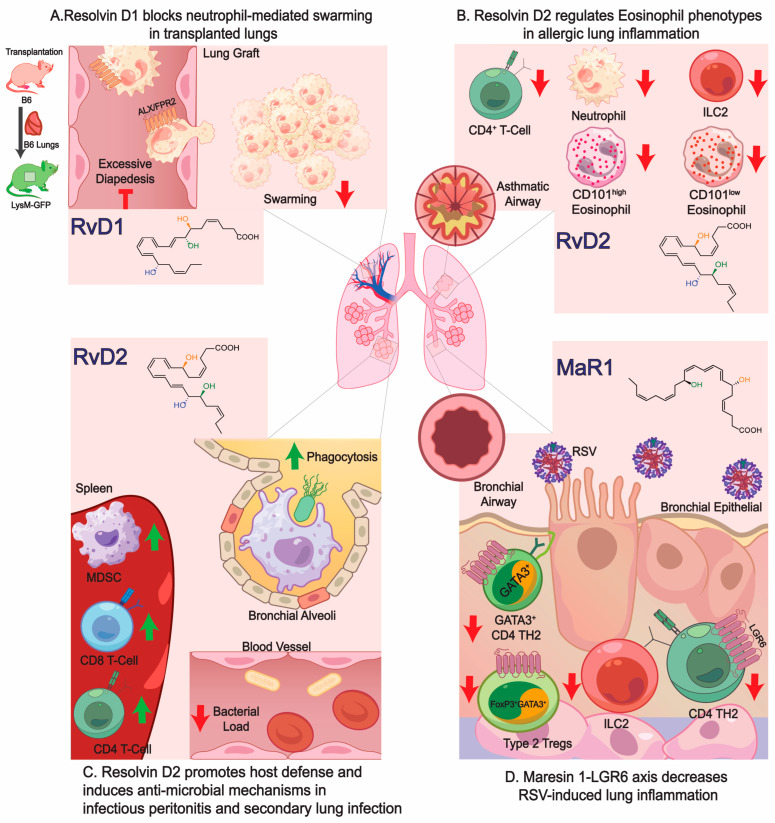
Role of SPMs in respiratory inflammation and injury. (**A**) In mouse lung transplantation, Resolvin D1 and its receptor, ALX/FPR2, block both excessive neutrophil diapedesis and swarming, demonstrating RvD1′s efficacy in preventing early neutrophil-mediated tissue damage after lung transplant [7]. (**B**) In allergic lung inflammation, RvD2 decreases the number of IL-5 producing CD4^+^ T-cells, ILC2 cells, and neutrophils while regulating the number of eosinophils [24]. (**C**) In a 2-hit model of sepsis with secondary lung infection, RvD2 promotes host defense and induces antimicrobial activity by decreasing bacterial load and increasing the number of MDSCs, CD8, and CD4 T-cells in the spleen [39,40]. (**D**) During RSV-induced lung inflammation, the activation of the MaR1-LGR6 axis reduces IL-13 secretion from ILC2 cells and a CD4 T helper while inhibiting FoxP3-expressing Tregs, highlighting the protective role of the MaR1-LGR6 signaling axis and leading to decreased viral burden, pathogen-induced inflammation, and the restoration of airway function [41].

Inflammation is a fundamental physiological response needed to restore tissue homeostasis [1,2]. A novel class of endogenous lipid mediators (LMs) that partially govern the resolution of inflammation are called specialized pro-resolving mediators (SPMs) [1]. SPMs are derived from omega-3 and 6-polyunsaturated fatty acids (PUFAs) that include arachidonic acid lipoxins, eicosapentaenoic acid (EPA)-derived (E-Series) [3] and docosahexaenoic acid (DHA)-derived Resolvins (D-Series) [4], Maresins [5], and Protectins [1,6]. SPMs regulate the resolution phase of acute inflammation by controlling excessive neutrophil trafficking into the inflammatory site. Recently, it was reported that Resolvin D1 (RvD1) blocks excessive neutrophil infiltration and swarming-mediated damage in a lung transplant model (Figure 1) [7]. SPMs also down-regulate pro-inflammatory mediators such as eicosanoids (prostaglandins (PG) and leukotrienes (LT)), chemokines (CXCL8 and CCL2), and cytokines (interleukins (IL-1β) and tumor necrosis factor alpha (TNF-α) [1,8]. In addition, SPMs enhance the macrophage efferocytosis of apoptotic neutrophils and cellular debris, which is the hallmark of the resolution of inflammation [9]. SPMs have shown their therapeutic effectiveness in pre-clinical models of acute and chronic inflammation, including atherosclerosis [8,10], neuroinflammatory diseases [11,12,13], cystic fibrosis [14], arthritis [15], ocular disease [16], liver disease [17,18], ischemia (Figure 1A) [19,20,21,22], asthma (Figure 1B) [23,24,25], infections (Figure 1C,D) [26,27,28], and cancer [29,30,31]. SPMs also regulate mechanisms involved in organ protection, wound healing, and tissue repair and regeneration, and they increase the host’s defense [1]. SPMs mediate their potent actions by activating specific G protein-coupled receptors on the surface of leukocytes and parenchymal cells. For example, Resolvin E1 binds to ChemR23, RvD1 binds to both the GPR32 and ALX/FRP2 receptors, while Resolvin D2 binds to GRP18 and Maresin-1 to the leucine-rich repeats-6 (LGR6). Of interest, both E-series and D-series precursors, 18-hydroxyeicosapentaenoic (HEPE) and 17-hydroxydocosahexaenoic acid (17-HDHA), respectively, are also bioactive mediators proven to be cardioprotective [32], promote the differentiation of IgE-secreting B cells [33], reduce exacerbated inflammatory responses against air pollution [34], and exhibit analgesic properties [35]. The stereochemical assignment, total organic synthesis, and biological actions of each of the D and E-series Resolvins, Protectins, and Maresins have been established [1,8,36,37,38], as reviewed in Table 1.

## 2. E-Series Resolvins

Charles Serhan discovered the first n-3 PUFA-derived Resolvins (Rvs)—RvE1, derived from EPA—whose structure was deduced to 5S,12R,18R-trihydroxy-6Z,8E,10E,14Z,16E-EPA [3]. This mediator is produced via transcellular biosynthesis from the interactions between human polymorphonuclear neutrophils (PMNs) and hypoxic vascular endothelial cells that release 18-HEPE, which is further converted by 5-lipoxygenase (LOX) by human PMN [3]. Using a chiral column liquid chromatography with tandem mass spectrometry (LC-MS/MS) analyses to examine RvE1 biosynthesis in human leukocytes and murine exudates, 18S-RvE1 was identified as a bioactive isomer of RvE1 [42]. A side-by-side comparison of both RvE1 and its isomer, 18S-RvE1, showed that both mediators were equally potent at enhancing the macrophage phagocytosis of zymosan, *E. coli*, and apoptotic neutrophils. In addition, both mediators reduced excessive neutrophil infiltration in a murine model of peritonitis, thus accelerating the resolution of inflammation [42]. In the nanogram to picogram ranges [43], RvE1 in vivo has potent actions that include clearing infections and stimulating resolution agonists in infections such as diabetes [89], tumor burden [31], colitis [90,91,92], periodontitis [3,54,93,94], lung inflammation [95,96,97], obesity [98], and atherosclerosis [99]. In primary cell cultures of nasal epithelia from cystic fibrosis patients, RvE1 restored a non-CF-like cilia beating phenotype, increased airway surface liquid layer height, and reduced the mucin MUC5AC thickness [100]. In cultured mouse dental pulp stem cells (mDPSCs), RvE1 facilitated AXin2-tdTomato^+^ cell proliferation, odontoblastic differentiation, and rescued impaired functions after lipopolysaccharide stimulation. In addition, RvE1 in this model reduced infection severity, prevented apical periodontitis, and accelerated the resolution of inflammation [101]. In a murine model of hypertension induced with angiotensin II, RvE1 potently lowered blood pressure, reduced aortic media thickness and infiltration of inflammatory macrophages and T cells while attenuating aortic fibrosis, and mitigated vascular smooth muscle cell transformation and proliferation [102]. RvE1 also improved severe aplastic anemia by increasing bone marrow macrophage efferocytosis and cellularity, platelet output, and survival in mice [103]. These findings demonstrate the importance of RvE1 actions in a wide range of diseases whose underlying condition was inflammation, which is mediated by leukocytes.

Other EPA-derived Resolvins have been identified in the exudate of mice with self-limited peritonitis, including RvE2, whose structure is 5S,18R-dihydroxy-6E,8Z,11Z,14Z,16E-EPA [42,54]. RvE2 is also found in plasma from healthy individuals [104]. Similar to RvE1, RvE2 can also be biosynthesized by hypoxic endothelial cells when interacting with human neutrophils under hypoxic conditions [54]. RvE2 stops neutrophil chemotaxis to IL-8 and stimulates membrane shape changes using a microfluidic chamber [53]. RvE3 (17R,18R-dihydroxy-5Z,8Z,11Z,13E,15E-EPA) containsa vicinal diol that also blocks neutrophil migration to the site of injury and is biosynthesized through the action of a 15-lipoxygenase (LOX) pathway [55]. RvE3 is more potent at decreasing depression behavior in mice when compared to RvE1 and RvE2 [57]. The newest member of the E-series Resolvins is RvE4, whose complete structure was deduced to be 5S,15S-dihydroxy-6E,8Z,11Z,13E,17Z-EPA via the activity of 15-LOX with EPA through lipoxygenation [58]. RvE4 was elucidated in physiologic hypoxic conditions that stimulated the clearance of senescent erythrocytes by macrophages, a process called erythrocytosis [58]. Of interest, during this process, EPA from erythrocytes is donated to macrophages to biosynthesize RvE4 [58]. Using targeted LC-MS/MS analysis and UV spectrophotometry, RvE4 was proven to be metabolized to the inactive state for 20-OH-RvE4. These metabolites have reduced bioactions in stimulating the macrophage efferocytosis of human senescent erythrocytes when compared to RvE4 [105]. In addition, RvE4 also stimulates the macrophage efferocytosis of apoptotic neutrophils and accelerates the resolution of hemorrhagic exudates in vivo in mice [58]. RvE4′s complete stereochemistry and total organic synthesis was achieved, allowing for its synthetic version to be used both as a standard for targeted LC-MS/MS and for further biological studies [59]. Importantly, RvE4′s biosynthetic route has been confirmed by independent investigators [106]. The stereochemical assignment, total organic synthesis, and biological actions of each of the E-series Resolvins have been established [1,8,36,37,38], as reviewed in Table 1.

## 3. D-Series Resolvins

DHA can be converted to give rise to the D-series Resolvins, which includes two separate pathways: 17S-RvDs and their aspirin-triggered (AT) epimers (17R-RvDs) [4]. These mediators are also potent agonists of the resolution of inflammation that control leukocyte functions, enhance the clearance of dead cells, and promote tissue repair without inducing immunosuppression [9]. The 17R Resolvins are biosynthesized from the 17R-hydroperoxy intermediate via acetylated COX-2, while the 17S-series via 15-LOX [4,107]. Five potent bioactive resolution agonists are further converted to RvD1, RvD2, RvD3, RvD4, and RvD5. RvD1, RvD2, and RvD5 are all biosynthesized in the initiation phase of the acute inflammatory response in vivo [1]. RvD1 (7S,8R,17S-trihydroxy-4Z,9E,11E,13Z,15E,19Z-DHA) and RvD2 (7S,16R,17S-trihydroxy-4Z,8E,10Z,12E,14E,19Z-DHA) are both produced from a transient 7(8)-epoxide Resolvin intermediate [66] via the 5-LOX [4]. RvD3 (4S,11R,17S-trihydroxy-5Z,7E,9E,13Z,15E,19Z-DHA) and RvD4 (4S,5R,17S-trihydroxy-6E,8E,10Z,13Z,15E,19Z-DHA) are biosynthesized by leukocytes during the resolution phase of inflammation [71] via the conversation of 4S, 5S-epoxy-Resolvin [108]. Collectively, these mediators are potent immunosolvents as they further stop neutrophil infiltration and transmigration to the site of inflammation and reduce pro-inflammatory cytokines and chemokines, while enhancing bacterial clearance and containing and accelerating macrophage efferocytosis [1,4,75].

Along those lines, RvD1 attenuates abdominal aortic aneurysm by decreasing immune cell infiltration, decreasing elastin fiber disruption, and increasing smooth muscle actin and aneurysm stability. Importantly, aortic aneurysm inflammation was increased in formyl peptide receptor 2 (FPR2) receptor-deficient mice, thus demonstrating RvD1/FPR2-dependent signaling [109]. This study demonstrated the RvD1/FPR2 signaling axis is fundamental for protection actions to accelerate the resolution of inflammation and organ injury [109]. RvD1 has been proven to accelerate the resolution of infectious inflammation by upregulating neutrophil and macrophage phagocytosis of pathogens [1,75]. Recently, RvD1 treatment attenuated Pseudomonas aeruginosa (PA) keratitis infected in mice by decreasing corneal bacterial loads and inhibiting excessive neutrophil infiltration, along with decreased TNF-α, IL-1β, and CXCL1. Importantly, in the cornea of these mice, RvD1 reduced M1 aggregation and enhanced M2 polarization, while increasing IL-10 and transforming growth factor-β [110]. Along these lines, corneal opacity development, thickening, and neutrophil infiltration were substantially reduced in RvD1 treatment in *S.aureus*-infected mice [111]. In a mouse model of sepsis-associated encephalopathy (SAE), RvD1 improved the learning and cognitive ability of SAE by inhibiting systemic and local inflammation in microglia via downregulation of NFκ-B, MAPK, and STAT3 signaling pathways [112]. RvD1 also protected mice from sepsis-induced kidney injury by improving mitochondrial function and reducing the apoptosis ration of kidney cortex cells [113]. RvD1 promoted bone regeneration via enhancement of osteoblast differentiation [114] and interstitial Siglec-macrophages [115]. For the first time, RvD1 production was reported to be induced in IgE-activated mast cells in vivo and vitro [25]. Taken together, these studies highlight the multi-faceted in vivo actions to dampen inflammation and promote the resolution of inflammation.

Another potent mediator of the D-series is RvD2 [1]. This resolution agonist reduces hepatic steatosis and fibrosis mediated by increasing the infiltration of reparative M2 macrophages and protection of reparative monocytes in the bone marrow [68]. Additionally, RvD3 improves impairment of insulin signaling in skeletal muscle and nonalcoholic fatty liver disease through AMPK by increasing phosph-AMPK expression and autophagy markers and alleviating insulin resistance, demonstrating its therapeutic effectiveness [116]. Importantly, RvD4 is also a strong regulator of neutrophils by controlling their deployment from the bone marrow after emergency granulopoiesis initiated by *E. coli* peritonitis [26]. RvD5 (7S-17S-dihydroxy-4Z,8E,10Z,13Z,15E,19Z-DHA) [117] also increases bacterial killing and clearance and is elevated in patients taking n-3 PUFA supplements via total parenteral nutrition (TPN) [76]. RvD5 plays a critical role in host defense and reduces arthritis by acting on T cells [77]. In type 1 diabetes mellitus animal models, RvD5 decreased anxious-like and depression behaviors and decreased pro-inflammatory cytokine IL-1β in the hippocampus and prefrontal cortex [118]. In female mice, RvD5 reduced infiltration of CD45^+^ hematopoietic cells into the kidneys, reduced activation of NFκB, and promoted the Nrf2 pathway by reducing Kelch-like ECH-associated protein 1 levels [119]. The stereochemical assignment, total organic synthesis, and biological actions of each of the D-series Resolvins have been established [1,8,36,37,38], as reviewed in Table 1.

## 4. Protectins

Within inflammatory exudates, DHA is also converted to conjugate triene structures, which include the pro-resolving mediators called Protectins (PD1). Protectins (10R,17S-dihydroxy-4Z,7Z,11E,13E,15Z,19Z-DHA), also named neuroprotection 1 (NPD1) in neural systems, are produced from the two omega-3 PUFA DHAs and n-3 docosapentaenoic acid [38]. Protectins’ biosynthesis is initiated through the 17-hydroperoxy (Hp)DHA [4], which is a biosynthetic product of human 15-LOX. PD1 and its epimoric positional isomer 17R-PD1/NPD1 are biosynthesized via an aspirin-acetylated COX-2 enzyme. The complete stereochemistry of these mediators has been established via nuclear magnetic resonance [120]. PD1 is identified in blood leukocytes, brain tissue, and glial cells. PD1 controls excessive neutrophil infiltration in vivo and pro-inflammatory cytokine production in human glial cells [38]. In a murine model of herpes simplex virus (HSV)-induced stromal keratitis (SK), topical administration of PD1 reduced the severity and prevalence of SK and new corneal neovascularization [78]. Further, PD1 reduced infiltration of neutrophils and pathogenic CD4^+^ T cells into the cornea and lowered the production of IFN-γ, IL-17, IL-6, CXCL1, CXCL10, VEGF-A, MMP-2, and MMP-9 in the corneas of infected animals. Importantly, PD1 increased the production of IL-10, demonstrating its valuable therapeutic approach to control SK lesions [78].

Along those lines, through the roles of transient receptor potential subtype V1 (TRPV-1) and TNF-α mediated spinal cord synaptic plasticity, PD1 blocked TRPV-1 and TNF-α evoked enhancement in synaptic transmission by inhibiting capsaicin-induced TRPV1 [121]. In a murine model of psoriasis, PD1 improved skin thickness, redness, and scaling by decreasing IL-1β, IL-6, IL-8, and IL-18BP gene expression, expression levels of CCL17, and inhibition of STAT1 and NF-κB signaling transduction pathways [122]. In zebrafish larva, PD1 improves fin fold regeneration and accelerates the resolution of inflammation without affecting the initial kinetics of neutrophil recruitment and the reverse transmigration potential [79].

## 5. Maresins

**Figure 2:**
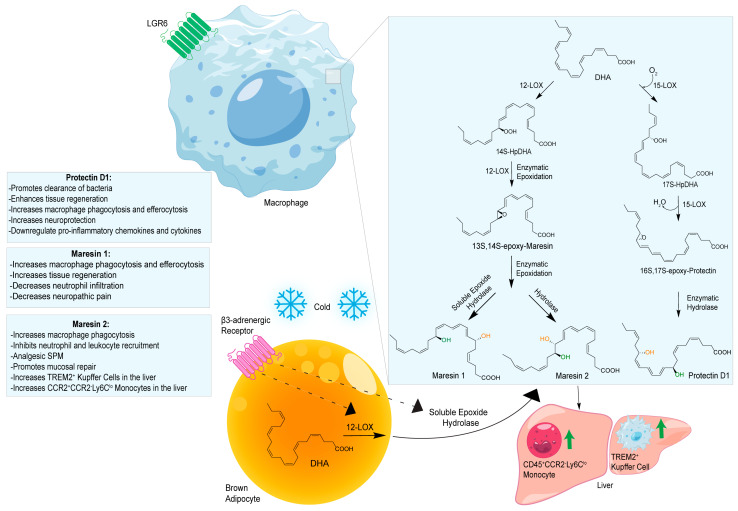
Biosynthetic Pathways of Protectins and Maresins in Macrophages and MaR-2 in Brown Adipose Tissue Activation. Right Side: The biosynthetic pathway of both Protectins and Maresins as derived in macrophages. Lower Left Corner: In obesity, thermogenesis activates brown adipose tissue (BAT) via cold-induced stimulation of β3-adrenergic receptors and triggers the upregulation of ALOX12 (12-lipoxygenase) and soluble epoxide hydrolase enzymes, leading to the biosynthesis of MaR-2. Bottom Right: In the liver, MaR2 significantly increases levels of both infiltrating CD45^+^CCR2^-^Ly6C^lo^ monocytes, triggering receptors expressed on myeloid cells-2 (TREM2)^+^ Kupffer cells [88].

Macrophages play an integral role in regulating the innate host response to local inflammation and are central in orchestrating processes such as neovascularization and wound healing. Another family of pro-resolving mediators derived from DHA, called Maresins (macrophage mediators in resolving inflammation), exert potent phagocyte-directed actions that include the inhibition of neutrophil recruitment and stimulation of macrophage efferocytosis [38] (Figure 2). Maresins’ biosynthesis in macrophages is initiated by 12-lipoygenation (12-LOX) from DHA producing 14S-HpDHA, the hydroperoxyl intermediate, which is further converted via enzymatic 13(14)-epoxidation [38,123]. This results in the introduction of a hydroxyl group and a double-bond rearrangement to form the stereochemistry of bioactive Maresin 1 (MaR1), which has been deduced to 17R,14S-dihydroxy-4Z,8E,10E,12Z,16Z,19Z-DHA [5] (Figure 2). Similarly, the 13S,14S-epoxy-MaR intermediate is also a precursor of Maresin 2 (MaR2) whose structure has been elucidated to 13R,14S-dihydroxy-4Z,7Z,9E,11E,16Z,19Z-DHA. This product of DHA biosynthesis by 12-LOX produces 14S-hydroperoxide that is converted to the 13S,14S-epoxy-MaR and finally converted by a soluble hydrolase into MaR2 [86] (Figure 2). MaR1 is also biosynthesized through platelet–neutrophil interactions that initiate organ protection [124]. MaR1 pro-resolving actions are complemented by its ability to stimulate tissue regeneration, reduce pain, limit neutrophil infiltration in murine models, and reduce inflammation and chemotherapy-induced neuropathic pain in mice [125] (Figure 2). Intravenous perioperative treatment with MaR1 10 minutes and 24 h post orthopedic surgery delayed the development of fPOP (mechanical and cold allodynia) [126]. In cultured rat conjunctival goblet cells, MaR1 increased high-molecular-weight glycoprotein secretion and intracellular Ca2^+^ ([Ca2^+^]i) [127]. In a prospective study on medication-naïve adolescents with first-episode major depressive disorder, MaR1 was negatively correlated with depression severity [82].

The second member of these macrophage-derived pro-resolving mediators is MaR2, whose pro-resolving actions, at 1 ng, reduce neutrophil infiltration in mice peritonitis by 40% and at 10 p.m. enhance human macrophage phagocytosis of zymosan by 90% [86]. MaR2 acts as an analgesic SPM in murine models by inhibiting neutrophil and leukocyte recruitment, nociceptor neuron TRPV1 and transient receptor potential ankyrin 1 (TRPA1) activation, and calcitonin gene-related peptide (CGRP) release [128]. Additionally, MaR2 inhibits lipopolysaccharide (LPS)-induced mechanical hyperalgesia inflammatory pain and changes in cytokines [128]. Further, MaR2 is a potent pro-reparative molecule that promotes mucosal repair in models of dextran sulfate sodium-induced colitis or biopsy-induced colonic mucosal injury [87]. Functional analysis revealed that MaR2 promotes mucosal wound repair by driving intestinal epithelial migration through the activation of focal cell–matrix adhesion signaling in primary human intestinal epithelial cells [87]. In various orofacial pain models, MaR2 delivered via medullary subarachnoid injection significantly reduced phases I and II of orofacial formalin test in rats [129]. MaR2 also prevented the development of facial heat and mechanical hyperalgesia in post-operative rats [129]. Additionally, in models of trigeminal neuropathic pain (CCI-ION), repeated MaR2 injections reversed facial heat and mechanical hyperalgesia while increasing both c-Fos^+^ and CGRP^+^ activated (nuclear pNF-κB) neurons in the trigeminal ganglion. This study shows MaR2′s potent and long-lasting analgesic effects in inflammatory and neuropathic pain of orofacial origin [129].

In models of diet-induced obese (DIO) mice, MaR2 derived from brown adipose tissue contributes to the cold-induced resolution of inflammation by targeting liver macrophages. This reduces the expression of *IL-18*, *Tlr2*, *Casp1*, and *IL-1β*, leading to an increase in the levels of both infiltrating CAD45^+^CCR2^-^Ly6C^lo^ monocytes and triggering receptors expressed on myeloid cells-2 (TREM2)^+^ Kupffer cells [88]. These results suggest MaR2 serves a protective role by modulating monocyte/macrophage populations in the liver during obesity [88]. Collectively, these studies demonstrate Maresin’s critical importance in the resolution of inflammation and highlight its therapeutic effectiveness in a broad spectrum of diseases. The stereochemical assignment, total organic synthesis, and biological actions of both Protectins and Maresins have been established [1,8,36,37,38], as reviewed in Figure 2 and Table 1.

## 6. Therapeutic Effectiveness of SPMs in Respiratory Inflammation and Injury

Respiratory inflammation is caused by pathogens or by exposure to toxins, pollutants, irritants, and allergens [130]. SPMs, in various murine models, have shown extensive therapeutic effectiveness in reducing respiratory inflammation and lung injury. Acute respiratory distress syndrome (ARDS) is a life-threatening condition characterized by increased permeability of the alveolar–capillary barrier and impaired alveolar fluid clearance [131]. RvE1 improves the clearance of the alveolar fluid and expression of phosphorylated AKT, SGK1, NEDD4-2, and alveolar ENaC and NKA in LPS-stimulated cells [131]. A deficiency of RvE1 receptors (Chemerin 23) and enhanced omega3 PUFA levels (*fat*-1 mice) affect lung–brain interactions during ARDS by altering profiles of glial activity markers [132]. Further, in allergic rhinitis (AR), RvE1, LTB_4_, and RvD1 serum levels were measured using an enzyme-linked immunosorbent assay, revealing RvE1 and LTB_4_ levels to be significantly higher in AR patients than in healthy patients [133]. This indicates that imbalanced RvE1 and LTB_4_ contribute to the defective airway inflammation-resolution and subsequent progression toward chronic inflammation [133]. Similarly, in a murine model, RvD1 increased the anti-inflammatory M2 phenotype, phagocytic function, and apoptosis of recruited macrophages via the FasL-FasR/caspase-3 signaling pathway [134].

Further, RvE3 attenuated allergic airway inflammation in house dust mite (HDM) by down-regulating IL-23 and IL-17 [56]. RvD2 also accelerated the resolution of a mouse’s TH2 inflammation evoked by HDM sensitization and potently regulated TH2 cytokine production and action in a DRV2 receptor-dependent manner [23]. In allergic lung inflammation, RvD2 decreased the number of IL-5 producing CD4^+^ T-cells, ILC2 cells, and neutrophils, while regulating the number of eosinophils (Figure 2) [24]. In a 2-hit model of sepsis with secondary lung infection, RvD2 promotes host defense and induces antimicrobial activity by decreasing bacterial load and increasing the number of MDSCs, CD8, and CD4 T-cells in the spleen (Figure 2) [39,40]. During viral infections, intranasal-administrated PD1 decreased post-infection lung eosinophils and attenuated the respiratory syncytial virus (RSV)-induced suppression of interferon-lambda in a mouse’s lung in vivo. PD1 also increased interferon-lambda expression in human bronchial epithelial cells in vitro [80]. RvD1 increased the frequency of memory CD8^+^ T cells and during reinfection presented a high viral load in the lung and lower antibody response in the serum, suggesting that RvD1 modulates the expression and phenotype of memory CD8^+^ T cells [80]. Similarly, in RSV inflammation, activation of the MaR1-LGR6 axis reduced IL-13 secretion from ILC2 cells and CD4 T helper, while inhibiting FoxP3-expressing Tregs, highlighting the protective role of the MaR1-LGR6 signaling axis and leading to decreased viral burden, pathogen-induced inflammation, and restoration of airway function (Figure 2) [41]. In Influenza A virus, PD1 decreased the number of cells positive for Influenza A virus NP protein, the expression of NP mRNA, and the replication of the highly pathogenic H5N1 influenza virus [135].

Acute lung injury (ALI) is associated with lung inflammation and excessive infiltration of neutrophils [83]. MaR1 accelerated the resolution of inflammation in LPS-induced ALI by decreasing excessive neutrophil infiltration, pathohistological changes, production of pro-inflammatory cytokines (TNF-α, IL-1β, IL-6), chemokines, pulmonary myeloperoxidase activity, and pulmonary edema [83,84]. Also, MaR1 accelerated caspase-dependent human neutrophils and the production of IL-10 [83]. In concanavalin A (ConA)-induced ALI, MaR1 improves liver functions and survival, and increases macrophage apoptosis [85]. MaR1 also attenuates the inflammatory response, hepatocyte apoptosis, histopathological damage, and reactive oxygen species (ROS) in macrophages [85]. In trauma hemorrhagic shock, MaR1 effectively alleviates lung injury by inhibiting the excitation of the TLR4/p38-MAPK/NFĸB pathways and suppressing IL-6 and TNF-α in BALF [136]. In ovalbumin (OVA), the administration of OVA^+^MaR2 reduces the number of inflammatory cells in BALF, levels of pro-inflammatory cytokines in serum, expressions of Caspase-1 proteins, and mucus secretion in lung tissue [137].

Along these lines, in asthma, exogenous MaR1 reduces lung inflammation and ILC2 expression of IL-5 and IL-13. MaR1 also increases amphiregulin and de nova generation of regulatory T cells, which further suppresses cytokine production in TGF-B-dependent [138]. During chronic bronchitis and obstructive pulmonary diseases, MaR1 decreases bronchoalveolar lavage neutrophil infiltration, IL-6, TNF-α, and CXCL1 levels [139]. Severe acute pancreatitis is an inflammatory disorder which progresses with local and systemic injury and is associated with a relatively high mortality rate. In cerulean and LPS-induced models, MaR1 decreased amylase, lipase, TNF-α, IL-1β, IL-6 in the serum, and myeloperoxidase activity both in the pancreas and lung tissues. This demonstrates MaR1′s ability to alleviate the inflammation of the pancreas and lung by inhibiting the activity of NFκ-B in experimental models [140].

Recently, SPMs have been identified in human peripheral blood and serum [141,142] and various organs and tissues using LC-MS/MS-based profiling by independent investigators around the world [143,144,145,146,147,148]. These include stenotic aortic valves [149], metabolic syndromes [150], nonobstructive coronary artery disease [151], human vagus nerve [152], multiple sclerosis [153], blister [154], chronic rhinosinusitis [155], and COVID-19 [156,157] employing deuterium-labeled synthetic standards for quantification within the range of pg/mL-ng/mL [158,159,160]. For example, in bronchoalveolar lavage fluid from COVID-19 patients, RvD3 and PD1 were increased along with pro-inflammatory lipids such as LTB_4_ and cysteinyl LTE_4_. Recently, PGE_2_ was identified in mild, moderate, and severe COVID-19 cases [161]. In contrast, RvD4 was identified to be associated with mild infections, RvD5 with mild and moderate cases, and MaR2 and RvE1 with severe cases [161]. Additionally, intravenous omega-3 supplementation in COVID-19 patients stimulated higher production of RvE3 compared with those that received the placebo [141,162]. Further, SPMs have also been identified in the cerebral spinal fluid of Alzheimer’s disease [163], serum [141,142], human atherosclerosis plaque [164,165], saliva of heart-failure [166], and plasma of peripheral artery disease [167] patients. All SPM standards are commercially available.

RvE1 was negatively correlated with a range of measures of adiposity in men and women, including those of smokers [168]. A population study of >978 individuals reported that RvE1 was significantly lower in individuals with obesity compared with those with a healthy weight [168]. Taken together, these studies suggest the importance of SPM production by human cells in response to COVID-19, viral infections, and chronic inflammatory conditions. Table 2 lists the endogenous levels of SPMs.

## 7. SPMs in Ischemic Injury

Neutrophil-mediated damage after ischemia reperfusion injury (IRI) plays a critical role in its pathogenesis by the increased collateral tissue and edema. Recently, RvD1 proved to potentially control excessive neutrophil recruitment, extravasation, and swarming that protected the lungs from ischemia-perfusion injury after transplantation in mice (Figure 2) [7]. Similarly, RvD1 reduced early pulmonary inflammation and protected neutrophil-mediated lung IRI. This protection was lost in FPR2 receptor-deficient mice, demonstrating that RvD1′s actions are mediated through signaling in its FPR2 receptor [176]. Along these lines, in a model of acute ischemic stroke (AIS), RvD1 reprogramed microglial energy metabolism to enhance neutrophil clearance and decrease AMPK-dependent oxidative damage and neural injury [177]. Importantly, increased serum levels of RvD1 in AIS patients are associated with improved neurological recovery and lower risks of recurrence and death [178]. In rats, MaR1 alleviates liver IRI by activating hepatocyte cell division, increasing IL-6 cytokine levels, and the nuclear localization of Nrf-2 [179]. Further, MaR1 ameliorates induced lung IRI by reducing myeloperoxidase, TNF, BALF leukocyte count, the expression of nuclear Nrf-2 and cytosolic HO-1 in lung tissue, ROS, methane dicarboxylic aldehyde, and 15-F2t-isoprostane generation [180].

Chronic inflammation plays an important role in the pathogenesis of ischemic renal injury (RI). PD1 decreases polymorphonuclear leukocyte recruitment, chemokines, cytokines, and pro-inflammatory eicosanoids while increasing renoprotective heme-oxygenase-1 protein in kidneys [81]. MaR1 protects against renal IRI inflammation by inhibiting the expression of TLR4, phosphorylated Erk, JNK, P38, and nuclear translocation of NFκ-B [181].

Along these lines, in hind limb ischemia (HLI), exogenous RvD2 enhances perfusion recovery by reducing neutrophil accumulation and the plasma levels of TNF-α and GM-CSF. RvD2 also enhances endothelial cell migration in a Rac-dependent manner via the activation of its receptor, GPR-18, and rescued defective revascularization in diabetic mice [182]. In a murine model of AIS, treatment of RvD2 ameliorated permanent middle cerebral artery occlusion-induced brain injury, neurological dysfunction, and inflammatory response. RvD2 also recused the resolution of inflammation by promoting macrophage/microglia polarization to pro-resolving M2 phenotype ex vivo and in vivo [183].

In hepatic IRI, MaR1 reduces ALT and AST levels, necrotic areas, inflammatory responses, oxidative stress, and hepatocyte apoptosis in the liver [184]. Akt signaling was increased in MaR1-treated IRI groups, demonstrating the importance of the Akt signaling pathway in the liver against hepatic IRI [184]. Also, MaR1 alleviates hepatic IRI by inhibiting NFκ-B activation and caspase-3/GSDME-mediated inflammatory responses [185].

In a hypoxic-ischemic (HI) brain injury, PD1 prevented the expansion of the ischemic core by 40% while improving coordination and motor abilities. At 7 days after HI injury, PD1 decreased ipsilateral hemisphere atrophy and preserved motor functions. Further, PD1 reduced mitochondrial BAX translocation and oligomerization, cytochrome C release, and apoptosis-inducing factor nuclear translocation [21]. This demonstrates PD1′s ability to preserve the mitochondrial membrane structure and reduce BAX mitochondrial translocation and activation [21]. Traumatic brain injury (TBI) is followed by secondary inflammation in the brain. PD1 decreases the lesion area at 72 h compared to no treatment in rats. Further, there are no differences in neuronal degeneration, apoptosis, anti-inflammatory enzymes, antioxidative enzymes, or immune cells, suggesting PD1 has minimal effects after focal penetration TBI and is beneficial for preventing brain tissue damage [186]. Cerebral ischemia-reperfusion injury is a common pathological feature in ischemia stroke. In PC12 cells induced by oxygen and glucose deprivation/reoxygenation (OGD/R), the pretreatment of PD1 protects cells against ischemia by enhancing cell survival and decreasing the levels of autophagy and oxidative stress markers. PD1 treatment also promotes the production of RNF146 and Wnt/B-catenin in cells following OGD/R experiments [187]. Further, in mice with brain ischemia reperfusion, MaR1 reduced pro-inflammatory mediators, NFκ-B p65 activation, the infarct volume and neurological defects, and nuclear localization to protect brain tissue and neurons from injury [188]. In a murine model of cerebral IR, MaR1 downregulated AC-NF-κB, BAX expression, pro-inflammatory factor levels (IL-1, IL-6, and TNF-α), neuronal degeneration, and the infarct size [189].

## 8. The Role of SPMs in Cancer

Inflammation is one of the hallmarks of cancer that contributes to tumor development and progression [190]. Unresolved inflammation can lead to fibrosis and enhance cellular senescence, untimely leading to cancer development and progression. SPMs have been reported to regulate intrinsic mechanisms within cancer cells, including decreasing their proliferation, enhancing cell death mechanisms, and increasing immune-mediated functions [65]. In murine models of cancer, SPMs have anti-cancer properties by decreasing tumor cell proliferation and by modulating tumor-associated macrophages (TAMs) to adopt a phenotype that is less immunosuppressive and more anti-tumoral [65]. Specifically, RvD1 and RvD2 are shown to suppress TAMs and enhance tumor cell debris, suggesting that SPMs can modulate macrophage polarization and increase efferocytosis, a fundamental function of SPMs [63,64]. Along these lines, RvD1, RvD2, and RvE1 heighten human monocyte-derived macrophages efferocytosis of cellular debris from chemotherapy-induced tumor cells and reduce the secretion of pro-inflammatory cytokines [31]. Additionally, SPMs (RvE1, RvD1, RvD2, RvD3, and RvD4) have been shown to reduce tumor growth in hepatocellular carcinoma [50,191], melanoma [31], oral squamous cell carcinoma [70], lung carcinoma [31,51,52], and pancreatic and prostate cancers [31,51]. Specifically, RvD2 shows in vitro and in vivo dose-dependent antitumor actions in oral squamous cell carcinoma [70]. Importantly, serum levels of RvD1 decrease in colorectal patients [192]. RvE1 in mice prevents liver injury and cancer cell transformation in hepatocellular carcinoma cells [50]. RvD2, RvD3, and RvD4 have also been demonstrated to reduce metastases in tumor-bearing mice of lung, liver, and pancreatic cancers [52]. Notably, the gene expression of D-series-resolving biosynthetic enzymes is suggested to be a predictor with better clinical outcomes in patients with head and neck squamous cell carcinomas [193]. MaR1 reduced UVB-induced skin edema, neutrophil recruitment, cytokine production, matrix metalloproteinase-9 activity, keratinocyte apoptosis, epidermal thickening, mast cell count, and degradation of skin collagen in hairless mice [30]. Taken together, these studies suggest that the biosynthetic pathways of SPMs, including their enzymes and receptors, provide a novel mechanism for SPMs as potent regulators of cancer development, progression, and metastasis included in experimental models. This highlights their potential therapeutic effectiveness for human cancer treatment.

## 9. Conclusions

The results reviewed herein demonstrate that SPMs are stereochemically defined potent resolution agonists that are now used worldwide in experimental models of human cells to elucidate their functions in controlling and accelerating the resolution of inflammation in both acute and chronic diseases. E-and D-series Resolvins have been reported by independent investigators around the globe to be effective and potent in modulating inflammation in diabetes, aging, liver fibrosis, colitis, periodontitis, lung inflammation, cystic fibrosis, hypertension, airway inflammation, asthma, organ transplantation, and depression behavior in mice. In addition, Resolvins enhance host defense to control, contain, and kill invading pathogens including *E. coli*, *S.aures*, Pseudomonas aeruginosa keratitis, and COVID-19, and to disengage infection-induced emergency granulopoiesis. Furthermore, Protectins demonstrate therapeutic potential in conditions such as herpes simplex virus-induced stromal keratitis and inflammatory pain, reducing severity, reducing inflammation, and promoting tissue regeneration by modulating cytokines and signaling pathways. Additionally, PD1 inhibits NP mRNA replication in Influenza A virus, preserves motor functions in brain injuries, and shows protective effects in models of traumatic brain injury and cerebral ischemia-reperfusion injury. Similarly, Maresins play a critical role in resolving inflammation and show therapeutic potential across various diseases, including respiratory conditions, liver injury, pancreatitis, and ischemia-reperfusion injuries in multiple organs. In cancer, SPMs demonstrate effectiveness in modulating macrophage polarization, enhancing efferocytosis, and reducing the secretion of pro-inflammatory cytokines. Taken together, these studies provide evidence that synthetic SPMs and their biosynthetic pathways, as well as the activation of their receptors, could provide a novel therapeutic avenue for the treatment of acute and chronic pathologies.

## Figures and Tables

**Table 1:** Complete Stereochemistry and Functions of E and D-series Resolvins, Protectins, and Maresins.

Resolvin	Structure and Complete Stereochemistry	Function
**Resolvin E1 (RvE1)**	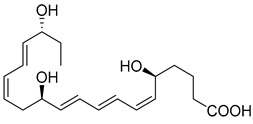 5S,12R,18R-trihydroxy-6Z,8E,10E,14Z,16E-EPA	-Enhances macrophage phagocytosis of zymosan, *E. coli*, and apoptotic neutrophils [42]. -Reduces excessive neutrophil infiltration in murine models [42]. -Clears infections and stimulates resolution agonists in various diseases [43]. -Reduces depression in mice [44,45]. -Stops PMN [3] and dendritic cell migration [46]. -Inhibits TRP Channels [47]. -Modulates T-cell response [47]. -Inhibits platelet aggregation [48]. -Reduces pro-inflammatory cytokines [49]. **Cancer:** -Prevents liver injury and cancer cell transformation in hepatocellular carcinoma cells [50]. -Inhibits tumor growth in lung, pancreatic, and prostate cancers [31,51,52].
**Resolvin E2 (RvE2)**	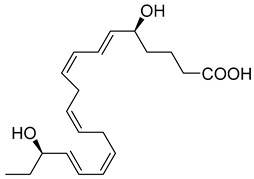 5S,18R-dihydroxy-6E,8Z,11Z,14Z,16E-EPA	-Stops neutrophil chemotaxis to IL-8 and stimulates membrane shape changes in microfluidic chamber [53]. -Decreases depression in mice [45]. -Stops PMN migration [42,54]. -Down-regulates leukocyte integrins [53].
**Resolvin E3 (RvE3)**	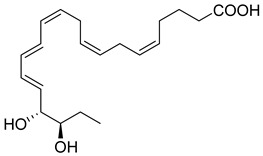 17R,18R-dihydroxy-5Z,8Z,11Z,13E,15E-EPA	-Blocks neutrophil migration to the site of injury [55]. -Reduces allergic airway inflammation in house dust mice by down-regulating IL-23 and IL-17 [56]. -Decreases depression in mice [57].
**Resolvin E4 (RvE4)**	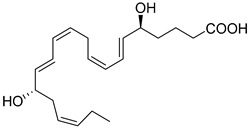 5S,15S-dihydroxy- 6E,8Z,11Z,13E,17Z-EPA	-Stimulates macrophage efferocytosis of apoptotic neutrophils in senescent blood cells [58,59]. -Accelerated resolution of hemorrhagic exudates in vivo in mice [58].
**Resolvin D1 (RvD1)**	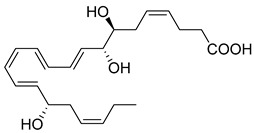 7S,8R,17S-trihydroxy-4Z,9E,11E,13Z,15E,19Z-DHA	-Stop neutrophil infiltration and transmigration to the site of inflammation [60]. -Reduces pro-inflammatory cytokines and chemokines [60]. -Accelerates macrophage efferocytosis [1]. -In mice, prevents neutrophil recruitment, extravasation, and swarming that protect lungs from ischemia perfusion injury after transplantation [7]. -Inhibits TRP channels [61]. -Modulates T cell response [62]. -Reduces IgE production in mast cells [25]. **Cancer:** -Increases human monocyte-derived macrophages efferocytosis of cellular debris from chemotherapy-induced tumor cells and reduces the secretion of pro-inflammatory cytokines [63,64]. - Inhibits tumor growth in lung, pancreatic, and prostate cancers [31,51,52]. -Suppresses TAMs and enhanced tumor cell debris [65].
**Resolvin D2 (RvD2)**	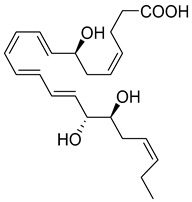 7S,16R,17S-trihydroxy-4Z,8E,10Z,12E,14E,19Z-DHA	-Stops neutrophil infiltration and transmigration to the site of inflammation [66]. -Reduces pro-inflammatory cytokines and chemokines [66]. -Accelerates macrophage efferocytosis [66,67]. -Controls hepatic steatosis and fibrosis mediated by increasing infiltration of reparative M2 macrophages and protection of reparative monocytes in the bone marrow [68]. -Inhibits TRP channels [47]. -Modulates T cell response [62]. -Suppresses NLRP3 inflammasome by promoting autophagy in macrophages [69]. **Cancer:** -Increases human monocyte-derived macrophages efferocytosis of cellular debris from chemotherapy-induced tumor cells and reduces the secretion of pro-inflammatory cytokines [63,64]. -Reduces metastases in tumor-bearing mice of lung, liver, and pancreatic cancers [52]. -Shows in vitro *and* in vivo dose-dependent anti-tumor effects in oral squamous cell carcinoma [70]. -Suppresses TAMs and enhances tumor cell debris [65].
**Resolvin D3 (RvD3)**	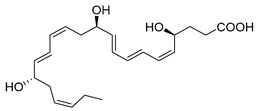 4S,11R,17S-trihydroxy-5Z,7E,9E,13Z,15E, 19Z-DHA	-Blocks PMN migration [71]. -Reduces pro-inflammatory cytokines and chemokines [72]. -Accelerates macrophage efferocytosis [71,72]-Restores epithelial barrier and function [71] **Cancer:** -Reduces metastases in tumor-bearing mice of lung, liver, and pancreatic cancers [52].
**Resolvin D4 (RvD4)**	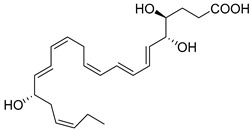 4S,5R,17S-trihydroxy-6E,8E,10Z,13Z,15E,19Z-DHA	-Controls neutrophil deployment from bone marrow after emergency granulopoiesis initiated by *E. coli* peritonitis [26]. -Enhances fibroblast phagocytosis [73]. -Enhance thrombosis clearance and decreases neutrophil extracellular traps [74]. **Cancer:** -Reduces metastases in tumor-bearing mice of lung, liver, and pancreatic cancers [52].
**Resolvin D5 (RvD5)**	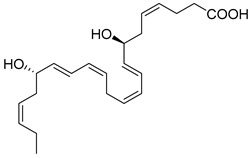 7S,17S-dihydroxy-4Z,8E,10Z,13Z,15E,19Z-DHA	-Enhances bacterial clearance [75]. -Accelerates macrophage efferocytosis [75]. -Elevated in patients taking n-3 PUFA supplements via TPN [76]. -Plays a critical role in host defense and reduces arthritis by acting on T cells [77].
**Protectin/** **NeuroProtectin 1** **(PD/NPD1)**	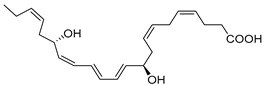 10R,17S-dihydroxy-4Z,7Z,11E,13E,15E,19Z-EPA	-Defends the host from viral infection and bacteria by killing and clearing microbes [38]. -Significantly reduces infiltration of neutrophils and pathogenic CD4^+^ T cells in HSV-induced SK [78]. -Induces macrophage polarization switch towards non-inflammation in Zebrafish larva fin fold regeneration [79]. -Decreases post-infection lung eosinophils in vivo in models of RSV [80]. -Decreases polymorphonucler leukocyte recruitment and chemokine, cytokine levels in IRI [81]. -Elevated in patients taking n-3 PUFA supplements via total parenteral nutrition (TPN) [76].
**Maresin 1** **(MaR1)**	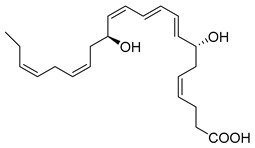 7R,14S-dihydroxy-4Z,8E,10E,12Z,16Z,19Z-DHA	-Negatively correlated with depression severity in medication-naïve adolescents with first-episode major depressive disorder [82]. -Exogenous MaR1-LGR6 axis decreases IL-13 production in FoxP3-expressing regulatory T cells [41]. -In ALI, accelerates the resolution of inflammation by attenuating neutrophil accumulation and pulmonary edema [83]. -Intratracheal injection of MaR1, in high doses, increases in pro-inflammatory cytokines, chemokines, and neutrophil infiltration in lung tissue [84]. -Attenuates hepatocyte apoptosis, ROS, and histopathological damage in macrophages [85]. -Elevated in patients taking n-3 PUFA supplements via TPN [76]. **Cancer:** -Reduces UVB-induced skin edema, neutrophil recruitment, cytokine production, and mast cells count in skin cancer [30].
**Maresin 2** **(MaR2)**	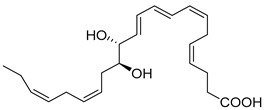 13R,14S-dihydroxy-4Z,7Z,9E,11E,16Z,19Z-DHA	-Reduces neutrophil infiltration in mouse peritonitis and enhances human macrophage phagocytosis of zymosan [86]. -Exogenous MaR2 promotes mucosal repair following dextran sulfate sodium-induced colitis [87]. -Modulates monocyte/macrophage populations in the liver of DIO mice [88]. -Elevated in patients taking n-3 PUFA supplements via TPN [76].

**Table 2:** Endogenous values of SPMs in various human tissues and organs.

Resolvins	Quantities in Tissue/Organ
**Resolvin E1** **(RvE1)**	**Plasma**: 2–22 pg/mL [169] **Stenotic Aortic Valves**: 500–3500 pg/g tissue [149] **Metabolic Syndrome (weight loss program):** 1339 ± 175 pg/mL [150] **Nonobstructive coronary artery disease (WARRIOR Trial)** [151] **Human Vagus Nerve:** 19.7 ± 12.6 pg/tissue [152] **COVID-19 and lung severity:** Severe: 112.6 pg/mL [161] **Obesity (adiposity):** Men: 6.5 pg/mL [168] Women: 5.2 pg/mL [168] **Salivary levels in Periodontal and cardiovascular therapies:** 0-6 months: 1.11–1.24 pg/mL [170] **Pregnancy:** First Trimester: 0.0049 ± 0.036 [171] Second Trimester: 0.048 ± 0.037 [171] Third Trimester: 0.024 ± 0.027 [171] ***Anetholea anisita* extract for scalp condition** [172]
**Resolvin E2** **(RvE2)**	**Obesity (adiposity):** Men: 10.7 pg/mL [168] Women: 11.4 pg/mL [168]
**Resolvin E3** **(RvE3)**	**Metabolic Syndrome (weight loss program):** 175 ± 44 pg/mL [150] **Obesity (adiposity):** Men: 19.2 pg/mL [168] Women: 15.9 pg/mL [168]
**Resolvin D1** **(RvD1)**	**Nonobstructive coronary artery disease (WARRIOR Trial)** [151] **Plasma:** 2–22 pg/mL [169] **Multiple Sclerosis:** 0.68 ± 0.32 pg/mL [153] **Synovial fluid:** 5 pmol/mL [173] **Blister:** 10–15 pg/mL [154] **Sputum (Cystic Fibrosis):** 200 pg/mL [174] **Chronic Rhinosinusitis** [155] **COVID-19 and lung severity:** Mild: 1.4 pg/mL [161] Severe: 1.0 pg/mL [161] **Obesity (adiposity):** Men: 7.4 pg/mL Women: 8.7 pg/mL [168] **Salivary levels in periodontal and cardiovascular therapies:** 0-6 months: 92.87–181.01 pg/mL [170] **Pregnancy:** First Trimester: 0.002 ± 0.001 [171] Second Trimester: 0.002 ± 0.001 [171] Third Trimester: 0.005 ± 0.011 [171]
**Resolvin D2** **(RvD2)**	**Nonobstructive coronary artery disease (WARRIOR Trial)** [151] **Metabolic Syndrome (weight loss program):** 27 ± 2 pg/mL [150] **Synovial fluid:** 5 pmol/mL [173] **Chronic Rhinosinusitis** [155] **COVID-19 and lung severity:** Mild: 9.1 pg/mL [161] Moderate: 9.1 pg/mL [161] Severe: 5.1 pg/mL [161] **Obesity (adiposity):** Men: 6.4 pg/mL [168] Women: 6.6 pg/mL [168] ***Anetholea anisita extract* for scalp condition** [172]
**Resolvin D3** **(RvD3)**	**Human Vagus Nerve:** 2.5 ± 0.7 pg/tissue [152] **Nonobstructive coronary artery disease (WARRIOR Trial)** [151] **Stenotic Aortic Valves:** 500–3500 pg/g tissue [149] **Blister:** 10–15 pg/mL [154] **COVID-19 and lung severity:** Mild: 1.5 pg/mL [161] **Obesity (adiposity):** Men: 5.2 pg/mL [168] Women: 5.1 pg/mL [168]
**Resolvin D4** **(RvD4)**	**Human Vagus Nerve:** 0.9 ± 0.3 pg/tissue [152] **Bone Marrow** [175] **COVID-19 and lung severity:** Mild: 0.5 pg/mL [161]
**Resolvin D5** **(RvD5)**	**Human Vagus Nerve:** 52.9 ± 20.2 pg/tissue [152] **Nonobstructive coronary artery disease (WARRIOR Trial)** [151] **Plasma:** 2–22 pg/mL [169] **Multiple Sclerosis:** 1.37 ± 0.43 pg/mL [153] **Synovial fluid:** 5 pmol/mL [173] **COVID-19 and lung severity:** Moderate: 15.0 pg/mL [161] Severe: 24.0 pg/mL [161] **Obesity (adiposity):** Men: 2.9 pg/mL [168] Women: 4.3 pg/mL [168]
**Protectin/NeuroProtectin D1** **(PD1/NPD1)**	**Human Vagus Nerve:** 82.7 ± 33.5 pg/tissue [152] **Multiple Sclerosis:** 0.14 ± 0.03 pg/mL [153] **Synovial fluid:** 5 pmol/mL [173] **Obesity (adiposity):** Men: 32.5 pg/mL [168] Women: 48.6 pg/mL [168] **Salivary levels in Periodontal and cardiovascular therapies:** 0-6 months: 101.2–146.67 pg/mL [170]
**Maresin 1** **(MaR1)**	**Human Vagus Nerve:** 6.9 ± 2.1 pg/tissue [152] **Nonobstructive coronary artery disease (WARRIOR Trial)** [151] **Metabolic Syndrome (weight loss program):** 35 ± 2 pg/mL [150] **Synovial fluid:** 5 pmol/mL [173] **COVID-19 and lung severity:** Mild: 36.7 pg/mL [161] Moderate: 40.9 pg/mL [161] Severe: 64.0 pg/mL [161] **Obesity (adiposity):** Men: 11.7 pg/mL [168] Women: 10.5 pg/mL [168] **Salivary levels in Periodontal and cardiovascular therapies:** 0-6 months: 125.51–337.03 [170] **Pregnancy:** First Trimester: 0.001 ± 0.001 [171] Second Trimester: 0.002 ± 0.001 [171] Third Trimester: 0.008 ± 0.020 [171]
**Maresin 2** **(MaR2)**	**COVID-19 and lung severity:** Mild: 5.5 pg/mL [161] Moderate: 3.0 pg/mL [161] Severe: 14.5 pg/mL [161]

## Data Availability

Not applicable.
